# Association of serum vitamin B_12_ levels with stage of liver fibrosis and treatment outcome in patients with chronic hepatitis C virus genotype 1 infection: a retrospective study

**DOI:** 10.1186/s13104-015-1248-z

**Published:** 2015-06-25

**Authors:** Nicolae-Catalin Mechie, Armin D Goralzcyk, Lars Reinhardt, Sabine Mihm, Ahmad Amanzada

**Affiliations:** Department of Gastroenterology and Endocrinology, University Medical Center Goettingen, Georg-August University Goettingen, Robert Koch Strasse 40, 37075 Göttingen, Germany; Division of Internal Medicine, Clinic of Herzberg and Osterode, Dr Froessel Allee, 37412 Herzberg am Harz, Germany

**Keywords:** Hepatitis C, Genotype 1, Vitamin B_12_, Sustained virological response

## Abstract

**Background:**

Chronic hepatitis C (CHC) is a global health challenge. New therapeutic agents with excellent sustained virological response (SVR) rates are available mainly in developed countries, while the majority of CHC patients live in countries with low health budget. Predictors of therapeutic response are therefore necessary. Vitamin B_12_ appears to be involved in hepatitis C virus replication.

**Methods:**

We therefore studied retrospectively the relationship between baseline serum vitamin B_12_ levels and clinical features in 116 CHC genotype 1 infected patients. Logistic regression models with univariate and multivariate analysis were used in the statistical analysis.

**Results:**

Baseline serum vitamin B_12_ levels were found to be positively associated with serum transaminase activities (AST, p = 0.002, ALT, p = 0.04), baseline viral load (p < 0.0001), stage of fibrosis (p = 0.0001) and favorable interferon-λ3/4 (IFNL3/IFNL4) rs12979860 genotypes (p = 0.04), and inversely with SVR (p < 0.001) as well as with rapid virological response (p = 0.001). Patients with baseline serum vitamin B_12_ levels below a cut-off value of 570 ng/L achieved a SVR rate of 59% with an odds ratio (OR) of 13.4 [confidence interval (CI) 4.3–41.9, p < 0.0001] compared to patients above the cut-off value. By combining serum vitamin B_12_ levels and IFNL3/IFNL4 rs12979860 genotypes, patients with baseline serum vitamin B_12_ levels below the cut-off value of 570 ng/L and IFNL3/IFNL4 rs12979860 CC genotype achieved a SVR rate of even 80% with an OR of 54 (CI 9.9–293, p < 0.0001) compared to patients above the cut-off value and non-CC-genotypes.

**Conclusion:**

Our data suggest baseline serum vitamin B_12_ levels as useful noninvasive marker for characterizing CHC patients. They might further help to identify responders to a standard treatment.

## Background

Patients with chronic hepatitis C virus (HCV) infection are at risk for progressive hepatic fibrosis, cirrhosis, portal hypertension, liver failure and hepatocellular carcinoma [1–4]. For the past decade, therapy with pegylated interferon-α (Peg-IFN-α) and ribavirin (RBV) yielded sustained virological response (SVR) rates of 40–50% among treatment naïve CHC patients with HCV genotype 1 infection [5, 6]. For those patients who did not achieve a SVR, retreatment options were limited to a re-exposure to the same medications, maybe modified in dose or duration. These retreatment strategies were accompanied by clinically significant morbidity (i.e. more pronounced side effects) and generally had a lower chance of resulting in a successful outcome [7, 8]. The recent approval of direct acting antiviral agents (DAAs) has inaugurated a new era in the treatment of CHC patients. These agents have raised the rates of SVR above 90% [9–16].

However, these clinical trials were performed in highly selected, triaged patients and the cost of antiviral therapy i.e. is only for sofosbuvir approximately 60,000 € [17]. This is especially relevant in view of most of CHC patients living in developing countries. Antiviral treatments consisting of sofosbuvir, ledipasvir, daclatasvir or simeprevir are not necessarily available in these countries yet. Thus, it is important to identify patients who do have a fair chance to respond to standard combination therapy, to avoid unnecessarily inducing detrimental side effects and to offer a treatment option, which is affordable for most of the patients. Several studies to date have aimed to identify accurate and sensitive predictors of treatment response. Besides HCV genotype, several other factors related to the virus [e.g. viral load at treatment initiation or rapid virological response (RVR)] and host (e.g. race, age, body weight, insulin resistance, serum lipids, fibrosis stage, serum ferritin concentration and genetic variations in the IFNL3/4 genes) have been shown to determine treatment-induced SVR in CHC patients [18–24].

HCV is a positive-sense, single-strand RNA virus that possesses an internal ribosomal entry site (IRES) at the 5′ terminus of its genome [25]. The IRES element is a complex RNA structure containing distinct domains which specifically interact with the ribosomal subunits and positions them directly over the initiation codon [26]. HCV IRES-mediated translation initiation is part of the viral replication mechanism and, given its specificity and sensitivity to minor structural changes, it is considered one of the targets for antiviral strategies. It has been shown in an in vitro system that vitamin B_12_ inhibits HCV IRES-dependent translation, probably by directly interacting with HCV IRES RNA [27, 28]. At the same time, vitamin B_12_ appears to be biologically significant for HCV replication, as high serum vitamin B_12_ levels were shown to be associated with high serum HCV-RNA levels in CHC patients [28]. A study by Rosenberg et al. [29] suggested high serum vitamin B_12_ levels to be favorable for achieving an end-of-treatment response in CHC patients. Accordingly, Rocco et al. [30] showed in an open-label pilot study that the addition of vitamin B_12_ to standard-of-care increases clearance of infection rates in treatment naïve CHC patients.

The aim of this study was to assess the relationship between serum vitamin B_12_ levels and clinical, histological features of CHC and to analyze its capacity as a predictor for sustained virus clearance upon a combination therapy with Peg-IFN-α and RBV.

## Methods

### Patients

A total of 116 CHC genotype 1 infected patients were included in this study and had their records reviewed. All 116 patients were from Germany and of Caucasian origin. CHC infection was defined by the presence of HCV-RNA in the blood for at least 6 months. Liver biopsy specimens were processed using standard techniques and evaluated for stage of fibrosis and grade of activity according to the established criteria [31]. All 116 patients were treated with dual antiviral therapy consisting of Peg-IFN-α and RBV and followed up at the Department of Gastroenterology and Endocrinology, University Medical Center of Goettingen, Germany. Patients with an active hepatitis B virus or human immunodeficiency virus infection, those with continued alcohol abuse or those who were receiving immunosuppressive medications were excluded. Increased serum levels of vitamin B_12_ can be seen in myeloproliferative disorders such as chronic myelogenous leucemia or primary polycythaemia, acute fulminant hepatitis, hypereosinophilic syndrome and sometimes in renal failure [32]. None of the patients included into the present study had a diagnosis of any of these conditions. All patients gave written informed consent to participate in the study in accordance with the ethical guidelines of the 1975 Declaration of Helsinki. The study was approved by the ethics committee of the University Medical Center (initial approval number 4/8/93 with subsequent amendments). Patients with CHC were treated either with Peg-IFN-α_2b_ at a dose of 1.5 µg/kg body weight in combination with weight-based ribavirin (800–1,400 mg per day) or 180 µg Peg-IFN-α_2a_ in combination with weight-based ribavirin (1,000 or 1,200 mg per day). Depending upon their individual tolerance and response parameters, both the dose and duration of treatment were adjusted. Serum HCV-RNA levels were monitored monthly. A rapid virologic response (RVR was defined as the elimination of viral RNA to a level below the limit of detectability (<50 copies/ml) during the first 4 weeks of therapy. Successful treatment was defined as a SVR, defined as the lack of detectability of HCV-RNA 6 months after cessation of therapy. The enzymatic activities of serum aspartate aminotransferase (AST), γ-glutamyl-transferase (γ-GT) and alanine aminotransferase (ALT) as well as baseline serum vitamin B_12_ levels were determined by utilizing the automated systems of the Central Laboratory of the Department of Clinical Chemistry at University Medical Center Goettingen.

### Isolation of genomic DNA and IFNL3/IFNL4 rs12979860 single nucleotide polymorphism (SNP) genotyping

These procedures were performed as described previously [33].

### Detection and determination of serum HCV-specific RNA and HCV genotype

Serum HCV-specific RNA was determined utilizing a nested RT-PCR assay and subsequent determination of the HCV genotypes. These procedures were performed as described previously [33].

### Statistical analyses

Associations between serum vitamin B_12_ levels with continuous (i.e., HCV viral load and serum ALT levels) and dichotomic variables (e.g., SVR versus no SVR, stage of liver fibrosis, hepatitis activity, and degree of steatosis) were assessed in logistic regression models, respectively. After univariate analysis, multivariate analysis was performed for significant associations. Multivariate analysis were obtained by using backward selection, with a p value >0.10 for removal from the model. Continuous and categorical variables were compared between those with a SVR and those without utilizing Wilcoxon Mann–Whitney, χ^2^ and Fisher’s exact tests. As our observational data regarding serum vitamin B_12_ levels were skewed we have decided to use quartiles, interquartile range (IQR) and Spearman’s correlations between continuous variables in our analysis. A p value of <0.05 was considered to be statistically significant. All statistical analyses were performed using the statistic program R cited at http://www.r-project.org and logistic regression calculators cited at http://statpages.org/logistic.html. Formulas with risk scores that best predicted the study’s other endpoints (marked fibrosis and cirrhosis) were constructed by entering different sets of independent variables into the regression model. Hardy–Weinberg equilibrium calculations cited at http://ihg.gsf.de/cgi-bin/hw/hwa1.pl were used as well. The receiver operating characteristics (ROC) curve and the area under the receiver operating characteristics (AUROC) were calculated by using GraphPad Prism 5.

## Results

A total of 116 CHC genotype 1 infected, treatment-naïve patients were included in this analysis (Table 1). 41% were female, their median age was 51 years (range 22–80). Patients had either HCV genosubtype 1a (29%) or 1b (67%) or had coinfection with both genosubtypes 1a + b (4%). The baseline enzymatic activities of AST, γ-GT and ALT as well as baseline serum vitamin B_12_ and HCV-RNA levels of all patients are presented in Table 1. A baseline histological evaluation of a liver biopsy was available in all patients. 32% (37/116) of the patients showed moderate/severe hepatitis activity. 23% (27/116) had severe fibrosis or cirrhosis. 49% (57/115) of the patients had steatosis above 5%. IFNL3/IFNL4 rs12979860 genotyping revealed a genotype distribution of 44:54:14 (CC:CT:TT) with a minor allele frequency of 0.37. Genotype distribution met the Hardy–Weinberg equilibrium (p = 0.64).

**Table 1:** Patient baseline characteristics

Male/female sex n (%)	68/48 (59%/41%)
Age [median (range)]	51 (22–80)
HCV subtype n (%)1a/1b/1a + b	34/77/5 (29%/67%/4%)
HCV-RNA level [median (IQR)] copies/mL	1.8 × 10^6^ (4.5 × 10^5^–6.2 × 10^6^)
AST [median (IQR)] U/L	44 (32–73)
ALT [median (IQR)] U/L	51 (32–93)
γ-GT [median (IQR)] U/L	50 (28–100)
Vitamin B_12_ [median (IQR)] ng/L	488 (339–727)
Hepatitis activity n (%)	
Mild	79 (68%)
Moderate/severe	37 (32%)
Fibrosis n (%)	
Absent/mild/moderate	89 (77%)
Severe/cirrhosis	27 (23%)
Steatosis n (%)	
0–5%	58 (50%)
6–100%	57 (49%)
Missing	1 (1%)
IFNL3/IFNL4 rs12979860 n (%)	
CC	44 (38%)
CT	54 (47%)
TT	14 (12%)
Missing	4 (3%)

Laboratory data are presented as mean and interquartile (IQR); number of cases are given in total and as a percentage; Baseline serum vitamin B_12_ levels were available for 107 patients.

### Quartile of baseline serum vitamin B_12_ levels with regard to treatment response, laboratory, histological and IFNL3/IFNL4 rs12979860 genotypes

**Figure 1:**
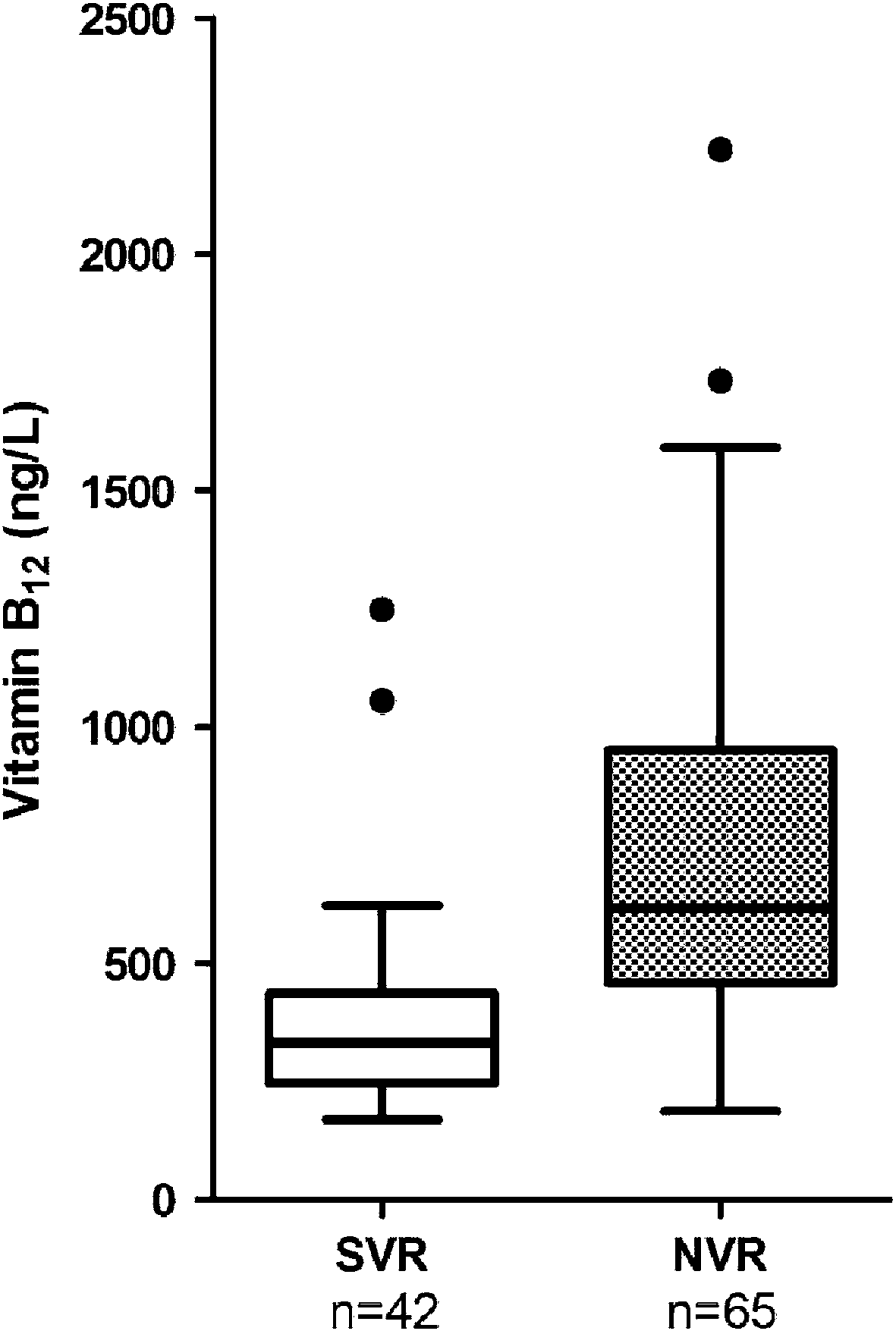
Association between baseline serum vitamin B_12_ levels and therapeutic outcome. Patients with sustained virological response (SVR) had lower baseline serum vitamin B_12_ levels than patients with non sustained virological response (NVR).

**Figure 2:**
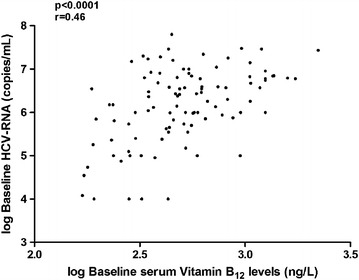
Spearman correlation between baseline serum vitamin B_12_ (ng/L) levels and HCV-RNA (copies/mL). This figure is the plot of the log of the HCV viral load as a function of the log of total serum vitamin B_12_ level.

The median value of baseline serum vitamin B_12_ levels was 488 ng/L (IQR, 339–727). No patient had baseline serum vitamin B_12_ levels below the lower normal limit. Median baseline serum vitamin B_12_ levels were 333 ng/L in SVR patients and 616 ng/L in non-responders (p < 0.0001) (Figure 1). Table 2 displays the associations between baseline serum vitamin B_12_ levels and several clinical and demographic variables, categorized according to the quartiles of vitamin B_12_. Low baseline serum vitamin B_12_ levels were significantly associated with RVR (p = 0.001) and SVR (p < 0.001). Low baseline serum vitamin B_12_ levels were also associated with low serum activity of AST (p = 0.002), ALT (p = 0.04), lower stages of fibrosis (p = 0.0001) and the favorable allele C of the IFNL3/IFNL4 rs12979860 SNP (p = 0.04). Moreover, baseline serum vitamin B_12_ levels were positively and significantly correlated with baseline serum HCV-RNA load (p < 0.0001) (Figure 2).

**Table 2:** Quartile of baseline serum vitamin B_12_ levels with regard to host and viral factors and treatment response

Characteristics	<340 (n = 27)	340–488 (n = 27)	488–727 (n = 26)	>727 (n = 27)	*P* value
Male sex n (%)	19 (70%)	20 (74%)	13 (50%)	13 (48%)	0.29
Age [median (range)]	47 (23–77)	53 (22–70)	51 (32–73)	51 (23–71)	0.74
HCV subtype n (%)					
1a	8 (29%)	12 (44%)	7 (27%)	6 (22%)	0.55
1b	18 (67%)	15 (56%)	18 (69%)	19 (70%)	
1a + b	1 (4%)	0	1 (4%)	2 (8%)	
RVR n (%)	19 (70%)	12 (44%)	9 (35%)	5 (19%)	0.001
SVR n (%)	22 (81%)	12 (44%)	7 (27%)	2 (7%)	<0.001
AST [median (IQR)] U/L	39 (30–54)	42 (32–51)	45 (36–77)	73 (53–121)	0.002
ALT [median (IQR)] U/L	46 (24–94)	44 (27–64)	55 (36–85)	66 (49–150)	0.04
γ-GT [median (IQR)] U/L	38 (28–87)	52 (24–103)	63 (41–136)	68 (28–142)	0.12
Hepatitis activity n (%)					
Mild	21 (78%)	18 (67%)	19 (73%)	15 (56%)	0.58
Moderate/severe	6 (22%)	9 (33%)	7 (27%)	12 (44%)	
Fibrosis n (%)					
Absent/mild/moderate	26 (96%)	22 (81%)	22 (85%)	11 (41%)	0.0001
Severe/cirrhosis	1 (4%)	5 (19%)	4 (15%)	16 (59%)	
Steatosis					
0–5%	21 (78%)	19 (70%)	19 (73%)	15 (56%)	0.26
6–100%	6 (22%)	7 (26%)	7 (27%)	12 (44%)	
Missing	0	1 (4%)	0	0	
IFNL3/IFNL4 rs12979860 n (%)					
CC	16 (59%)	11 (41%)	5 (19%)	9 (33%)	0.04
CT	8 (30%)	15 (55%)	15 (58%)	12 (44%)	8 (30%)
TT	2 (7%)	1 (4%)	5 (19%)	5 (19%)	2 (7%)
Missing	1 (4%)	0	1 (4%)	1 (4%)	1 (4%)

### Treatment response with regard to baseline serum vitamin B_12_ levels, histological features, baseline and on-treatment HCV-RNA levels and IFNL3/IFNL4 rs12979860 genotypes

The overall SVR rate of CHC genotype 1 patients was 41%. Factors which were found to be associated to SVR in univariate analysis included low baseline serum vitamin B_12_ levels (p < 0.001), low stage of fibrosis (p = 0.01) and low degree of steatosis (p = 0.02), low baseline HCV-RNA levels (p < 0.001), RVR (p < 0.001) and IFNL3/IFNL4 rs12979860 CC genotype (p = 0.0001) (Table 3). A multivariate analysis revealed that all these parameters were significantly and independently related to sustained virus eradication (Table 3).

**Table 3:** Uni- and multivariate analysis of factors associated with treatment response

Characteristics	Univariate analysis		Multivariate analysis
		*P* value	*P* value
Male sex n (%)	68 (59%)	0.07	0.19
Age [median (range)]	51 (22–80)	0.28	
RVR n (%)	40 (82%)	<0.001	<0.001
Vitamin B_12_ [median (IQR)] ng/L	488 (339–727)	<0.001	<0.001
HCV-RNA level [median (IQR)] copies/mL	1.8 × 10^6^ (4.5 × 10^5^–6.2 × 10^6^)	<0.001	<0.05
IFNL3/IFNL4 rs12979860 CC n (%)	28 (64%)	0.0001	<0.001
Hepatitis activity n (%)			
Mild	79 (68%)	0.70	
Fibrosis n (%)			
Absent/mild/moderate	89 (77%)	0.01	<0.05
Steatosis n (%)			
0–5%	58 (50%)	0.02	0.01

*RVR* rapid virological response.

**Figure 3:**
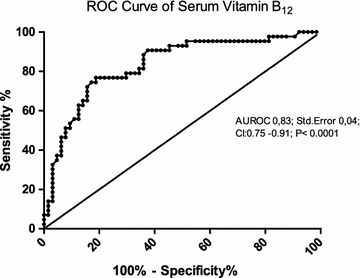
Receiver operating characteristics (ROC) Curve of baseline serum vitamin B_12_ levels. This ROC curve shows the relation between sensitivity and specificity regarding the baseline serum vitamin B_12_ levels and HCV therapy response.

**Figure 4:**
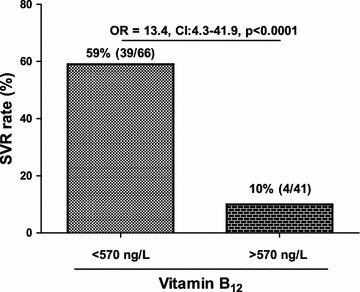
Association of serum vitamin B_12_ levels with a 570 ng/L cut-off value with regard to SVR. This *box plot* shows the relationship between baseline serum vitamin B_12_ levels below or above the cut-off value of 570 ng/L and the sustained virological response (SVR) rate. The *numbers in brackets* represent: number of patients with SVR/total number of patients.

**Figure 5:**
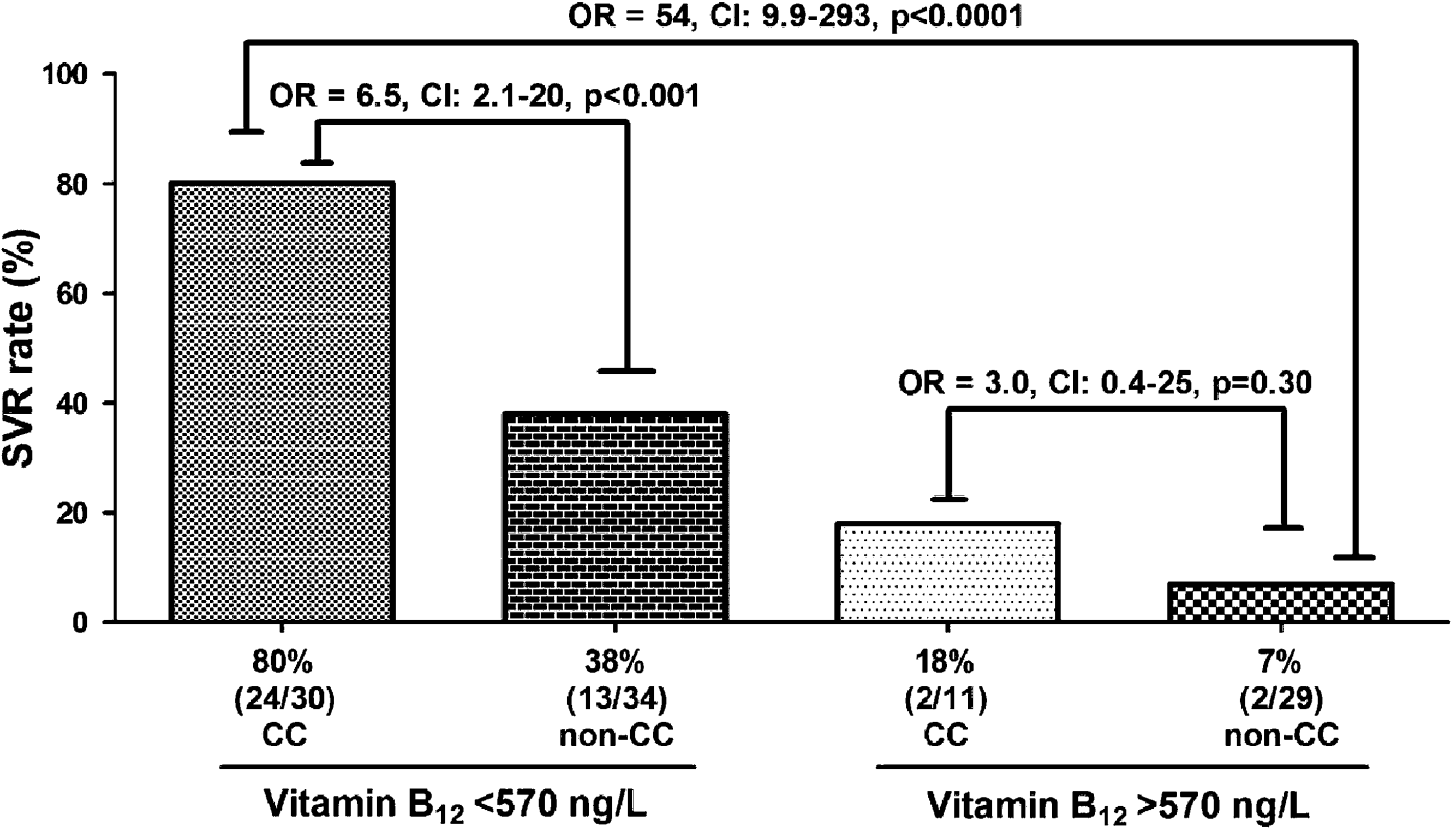
Association of IFNL3/IFNL4 rs12979860 genotypes and baseline serum vitamin B_12_ levels with a cut-off value of 570 ng/L with regard to SVR. This *Box plot* shows the SVR rates for antiviral therapy when both IFNL3/IFNL4 rs12979860 and baseline serum vitamin B_12_ levels below or above the cut-off value of 570 ng/L were considered. The *numbers in brackets* represent: number of patients with SVR/total number of patients in that group.

A cut-off value for serum vitamin B_12_ of 570 ng/L has been chosen using a ROC analysis with an AUROC of 0.83 (Figure 3). The sensitivity, specificity and positive and negative predictive values (PPV and NPV) of baseline vitamin B_12_ level were calculated to amount to 91, 58, 59 and 90%, respectively (data not shown). Patients with baseline serum vitamin B_12_ levels <570 ng/L achieved a SVR rate of 59% (39/66) with an OR of 13.4 (CI 4.3–41.9, p < 0.0001) compared to the group of patients with levels above 570 ng/L who achieved only a SVR rate of 10% (4/41) (Figure 4). Patients with baseline serum vitamin B_12_ levels below the cut-off value of 570 ng/L and IFNL3/IFNL4 rs12979860 CC genotype, however, achieved a SVR rate of 80% (24/30) with an OR of 54 (CI 9.9–293, p < 0.0001) when compared to patients above the cut-off value carrying the non-CC IFNL3/IFNL4 rs12979860 allele (Figure 5).

## Discussion

The therapy of CHC patients is currently undergoing a dramatic upheaval, especially with the introduction of the new DAAs such as Sofosbuvir, Simeprevir, Daclatasvir or Ledipasvir. With these new therapy regimens, patients achieve SVR rates above 90% [14–16, 34]. For these patients, there is not necessarily a need for new predictors. However, considering the situation worldwide, it becomes clear that only a fraction of CHC patients would have access to such an expensive therapy. The majority of patients living in countries with lower health budget might be treated with Peg-IFN-α and RBV in the next years, which is less effective and may have serious side effects. To optimize such an antiviral therapy regimen, more factors have to be evaluated and validated to protect patients from severe adverse events or discontinuation of therapy and to predict the individual probability of SVR with highest possible certainty.

There are a number of demographical, laboratory, histological, genetical and virological predictors for the treatment of CHC patients with Peg-IFN-α and RBV [21, 22, 35, 36] or with Peg-IFN-α, RBV and one of the first-generation protease inhibitors [37]. These factors could predict very accurately an individual’s chance to achieve a SVR. By combining independent predictors, a better prediction can be made. One example for this is the combination of IFNL3/IFNL4 rs12979860 genotypes and the ratio of γ-GT and ALT serum activities (γ-GT/ALT) [22, 38]. Simple and quickly determinable laboratory parameters may help physicians in countries with lower health budget to identify CHC patients who would achieve a high SVR rate upon an antiviral treatment with Peg-IFN-α and RBV.

There is some evidence that vitamin B_12_ inhibits dose-dependently the HCV IRES-dependent translation [28, 39]. On the other hand, high levels of serum vitamin B_12_ are statistically correlated with high viral load [28]. Vitamin B_12_ thus might have opposing effects on HCV translation and replication.

According to Lott et al. [28] HCV may have evolved to use high vitamin B_12_ levels in the hepatocytes for obtaining maximum replications values.

Several liver diseases such as hepatitis, cirrhosis, hepatocellular carcinoma and metastasis, may be accompanied by relative vitamin B_12_ deficiency secondary to impaired liver storage. This consequents to the increased release during hepatic cytolysis and/or decreased clearance by the affected liver [40]. Therefore, in this situation and given the natural role of vitamin B_12_ in the regulation of the HCV replication cycle [28], it is conceivable that administration of vitamin B_12_ might improve the rates of virological response to antiviral therapy in HCV carriers.

This study confirms the results of Lott et al. [28] with regard to a positive relationship between serum vitamin B_12_ levels and serum viral load. Furthermore, it also could be shown that serum vitamin B_12_ levels were associated with the stage of fibrosis in CHC-genotype-1-infected patients. In this context, the content of vitamin B_12_ concentration in the liver would be interesting, and possibly reduced. Moreover, this study demonstrated that vitamin B_12_ is associated with RVR and SVR and thus might be a further simple and quickly determinable predictor for antiviral treatment response to a regimen consisting Peg-IFN-α and RBV.

In contrast to these results, Rosenberg et al. [29] showed that higher baseline serum vitamin B_12_ levels were correlated with End-of-Treatment Response but not with SVR. However, the study of Rosenberg et al. [29] included 45 CHC genotype-1-infected patients only and therefore it may be statistically underpowered.

Recently, Rocco et al. [30] conducted the first prospective study, which showed that supplementation of vitamin B_12_ to Peg-IFN-α and RBV significantly improved the SVR rate compared to a control group without supplementation of vitamin B_12_. One reason for the better results of patients treated with Peg-IFN-α, RBV and vitamin B_12_ may be that vitamin B_12_ inhibits HCV IRES-dependent translation, probably by directly interacting with HCV IRES RNA [27, 28]. Another reason might be a modulating effect of vitamin B_12_ on the immune system [41].

The main limitation of this study is the observational nature regarding the analyzed data which cannot offer information about the molecular pathway how serum vitamin B_12_ predicts SVR.

## Conclusion

Baseline serum vitamin B_12_ levels were found to associate with the stage of fibrosis in CHC patients with HCV genotype 1 infection. Serum vitamin B_12_ levels were also found to independently predict sustained viral clearance to a combination therapy consisting of Peg-IFN-α and RBV. By combining the predictive value of IFNL3/IFNL4 rs12979860 genotype and serum vitamin B_12_ levels, discrimination of responding and non-responding individuals can reach an OR of 54 at best. The determination of serum vitamin B_12_ levels thus may be useful as a noninvasive surrogate marker for the stage of fibrosis on one hand and may also help to predict responsiveness to Peg-IFN-α and RBV therapy on the other.
